# MARK4 contributes to cilia formation by regulating the degradation of inhibitory OFD1 from the centriolar satellites

**DOI:** 10.1186/2046-2530-4-S1-P27

**Published:** 2015-07-13

**Authors:** R Carvalho, P Wiedemann, G Pereira

**Affiliations:** 1 Deutsches Krebsforschungszentrum, Heidelberg, Germany; 2 Hochschule Mannheim, Mannheim, Germany

## 

Cilia formation starts at the mother centriole and requires the activity of the microtubule affinity regulator, MARK4, which promotes axoneme extension during the initial phase of ciliogenesis. The defective axoneme extension in MARK4 depleted cells could be in part rescued by co-depletion of the inhibitory complex CP110/Cep97. Whether MARK4 only influences CP110/Cep97 or influences additional inhibitory components is not known. Interestingly, MARK4 has been recently shown to regulate the position and movement of autophagic vesicles within the cell. Recent studies also revealed that autophagy promotes ciliogenesis by inducing the selective degradation of the centriolar satellite pool of OFD1, an inhibitor of centriole elongation and axoneme extension. Here, we investigated whether MARK4 is functionally linked to autophagy to promote cilia formation. Analysis of RPE1 cells stably expressing YFP-LC3 shows that autophagosomes exhibit a perinuclear clustering upon MARK4 depletion. Quantitative immunofluorescence analysis shows that in MARK4 depleted cells, OFD1 levels at the centriolar satellites are higher than in control cells. This could be reverted by partial knock down of OFD1. By co-depleting MARK4 and OFD1, the cilia loss phenotype of MARK4 knockdown was partially reverted. Our results suggest that MARK4 acts in ciliogenesis by regulating the movement and position of the autophagosomes that degrade OFD1 at the centriolar satelites.

